# Autologous Fat Breast Reconstruction with Implant Completion-Experience with 29 Consecutive Cases and 33 Breasts

**DOI:** 10.1007/s00266-024-04042-w

**Published:** 2024-05-08

**Authors:** Yoav Gronovich, Ron Skorochod, Adi Maisel-Lotan

**Affiliations:** Department of Plastic and Reconstructive Surgery, Shaare Zedek Medical Center, Faculty of Medicine, Hebrew University of Jerusalem, POB 3235, 9103102 Jerusalem, Israel

**Keywords:** Breast reconstruction, Brava, Autologous fat transfer, Implants

## Abstract

**Background:**

Implant-based breast reconstruction is the most prevalent form of breast reconstruction. Autologous fat grafting (AFG) was conceptualized as an alternative to implant-based breast reconstruction and was found to be a reliable reconstruction modality. However, usually, a few grafting rounds are required to create the desired shape and size of the breast. Current literature describes ample experience with AFG as an adjunct to implant-based breast reconstruction for improving appearance. However, the utilization of breast implants following initial AFG has been sparingly described. The primary advantage of this study is the creation of new fat tissue as a breast mound. The reconstruction is then concluded by inserting an implant into this new mound. This approach reduces the overall number of fat injections needed to achieve the desired outcome, as well as the total volume of the implant.

**Methods:**

This IRB-approved retrospective study was conducted between January 2015 and December 2021. All women who underwent delayed breast reconstruction with AFG during this timeframe and wanted to complete it with a silicone implant as a last stage were included in the study.

**Results:**

A total of 29 patients (33 breasts) underwent delayed breast reconstruction with AFG and a silicone implant as the final stage. In all cases, the results were satisfying without any major complications. Minor complications were observed with one patient and included an infection.

**Conclusions:**

The findings of this study have demonstrated the effectiveness of this procedure together with patient satisfaction, thus highlighting the potential advantages that this approach offers.

**Level of Evidence III:**

This journal requires that authors assign a level of evidence to each article. For a full description of these Evidence-Based Medicine ratings, please refer to Table of Contents or the online Instructions to Authors www.springer.com/00266.

**Supplementary Information:**

The online version contains supplementary material available at 10.1007/s00266-024-04042-w.

## Introduction

Breast reconstruction procedures and approaches have evolved tremendously over the last decade. The introduction of novel concepts in the surgical and oncological treatment of benign and malignant breast lesions, alongside the improved prognosis of breast cancer patients, have put an emphasis on quality-of-life improvement associated with breast reconstruction in its various forms [1–3]. Breast reconstruction is often classified based on the timing of the procedure in relation to the surgical resection of the breast: immediate or delayed. Recently, the surgical paradigm has shifted toward immediate breast reconstruction, mainly due to its superior aesthetic outcome, lower financial cost, and improved patient satisfaction. Additionally, it contributes to psychological well-being during oncological treatment and rehabilitation [4–8]. However, delayed breast reconstruction is still encountered, mainly due to an individual surgeon's preference, significant patient comorbidities, planned post-mastectomy radiation therapy, or in patients emotionally not ready to discuss reconstruction options [9, 10]. Implant-based breast reconstruction is the most prevalent form of breast reconstruction in the United States. This may partially be explained by the greater patient morbidity, financial burden, and increased resource requirements associated with autologous and microsurgical reconstruction. However, in patients undergoing delayed breast reconstruction, implantation can be potentially problematic and typically requires prior tissue expansion [11, 12].

Additionally, implant-based reconstruction is often neglected in favor of autologous reconstruction in patients with a history of post-mastectomy radiation therapy, as it raises the risk for complications and adverse events [13–16]. Autologous fat grafting (AFG) for breast reconstruction was conceived as an alternative to reconstructing breasts with silicone implants. It was banned in 1987 because of unpredictable oil cyst formations that could not be differentiated from malignant lesions [17, 18]. Improvement of radiological diagnosis and surgical management resulted in the reconstitution of AFG as a valid option in breast reconstruction [19]. Historically, breast reconstruction with AFG has suffered from unpredictable rates of graft survival, that were found to decrease significantly when larger volumes of fat were grafted [20]. To overcome these inherent limitations, the use of a bra-like external tissue expander (BRAVA) before AFG, was introduced. The BRAVA expansion causes a marked temporary increase in breast size and generates a fibrovascular scaffold that improves graft survival in large volume AFG‘s [21–23]. The use of AFG was found to be a reliable independent reconstruction modality, establishing its versatility as an adjunct to improving the aesthetic outcome of traditional breast reconstruction procedures [24]. Current literature describes ample experience with autologous fat grafting for improving breast contour during initial implant-based breast reconstruction or as a means to correct the aesthetic appearance in the post-operative period However, the utilization of breast implants following initial AFG has been sparingly described.

The primary advantage of this concept is the creation of a new fat tissue as a breast mound. The reconstruction is then concluded by inserting an implant into this new mound. This approach reduces the overall number of fat injections needed to achieve the desired outcome, as well as reducing the total volume of the implant.

In this study, we aim to describe our experience with implant insertion in a new fat plane, as a supplementary procedure to initial AFG breast reconstruction.

## Methods

This retrospective study was conducted between January 2015 and December 2022. An Institutional Review Board approval was obtained (012-15 SZMC).

All women who underwent delayed breast reconstruction with AFG during this timeframe and wanted to complete it with a silicone implant as a last stage were included in the study.

### Sequence of Intervention

1. BRAVA—Use of the BRAVA device was suggested to all women undergoing delayed reconstruction who wanted to reconstruct their breast with autologous fat and was mandatory for patients who had undergone previous radiation therapy. Those who agreed, had to use the BRAVA for a period of 180 h before each AFG procedure. All other patients had the AFG without any pre-op preparation.

(2) AFG—The number of sessions was determined according to the patient's wishes, in order to get a symmetrical result (for unilateral reconstruction) and desired size and shape (for bilateral reconstruction). The interval between each session ranged from 3 to 6 months.

*The technique*: Aseptic irrigation was performed. A tumescent compounded of NaCl and Epinephrine 1:1,000,000 was injected to the abdominal wall. Autologous fat was harvested using the Lipografter^©^ system. After dissemination for 15 minutes, the fat was injected using a 3 ml syringe through the same system. Ribbons of fat were delivered to the chest bed in the sub-dermal plane, the pectoralis muscle plane and the sub-pectoral plane. The limit for AFG delivery was determined by the capacity of the tissue and the resulting aesthetic appearance (video 1).

(3) Silicone implant insertion—patients who decided to conclude the reconstruction with a silicone implant (for both the reconstructed breast and the healthy breast or the reconstructed breast only) went through this operation at least 6 months from the last AFG procedure.

*The technique*: Aseptic irrigation was performed. A 4–5 cm incision at the IMF was made and a dissection was performed to the fascia of the pectoralis muscle. Subsequently, a neo-fat plane above the pectoralis muscle was established, mirroring the sub-glandular plane found in a healthy breast. A sizer implant was inserted to that plane in order to define the size of the implant. The pocket was then irrigated, and a silicone implant was inserted. The incision was closed in layers. The other breast was operated on in the same way, if necessary (video 2).

Patients were followed up on POD 1, 7, 14, after 3, 6 and 12 months following the last surgery. Each follow-up included a physical examination and digital photography. All patients underwent ultrasonography before reconstruction and after AFG sessions. Patients were asked to rate their satisfaction on a *Likert type scale* (scale from one to five).

### Statistical Analysis

Analysis of the study sample was carried out using Microsoft Excel version 16.0 for Windows.

## Results

**Fig. 1:**
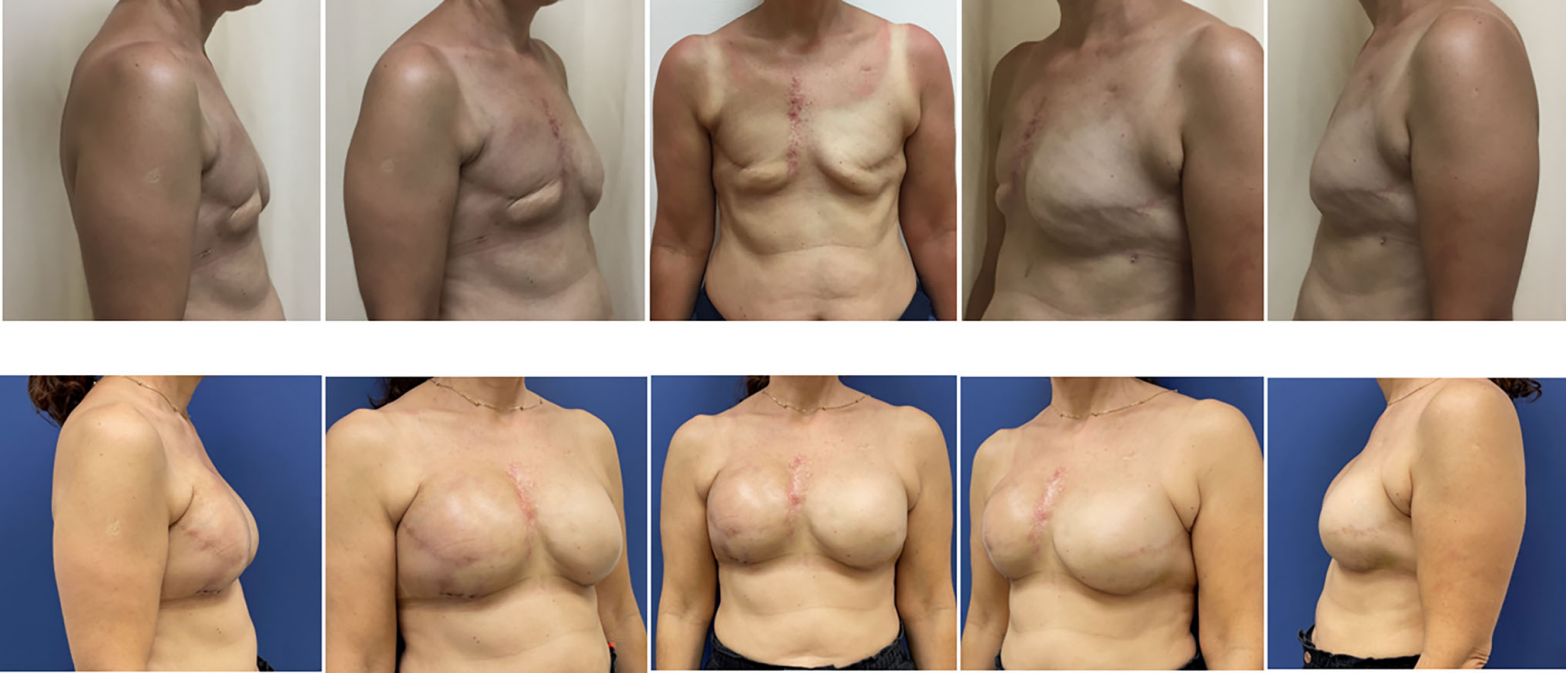
Above-after bilateral mastectomy and post-mastectomy radiation to right breast. Below-after 3 rounds of AFG to left breast, 5 rounds of AFG to right breast (average of 200 cc per round per breast), and conclusion with implant in neo-fat plane (Mentor, round moderate-plus 275 cc)

**Fig. 2:**
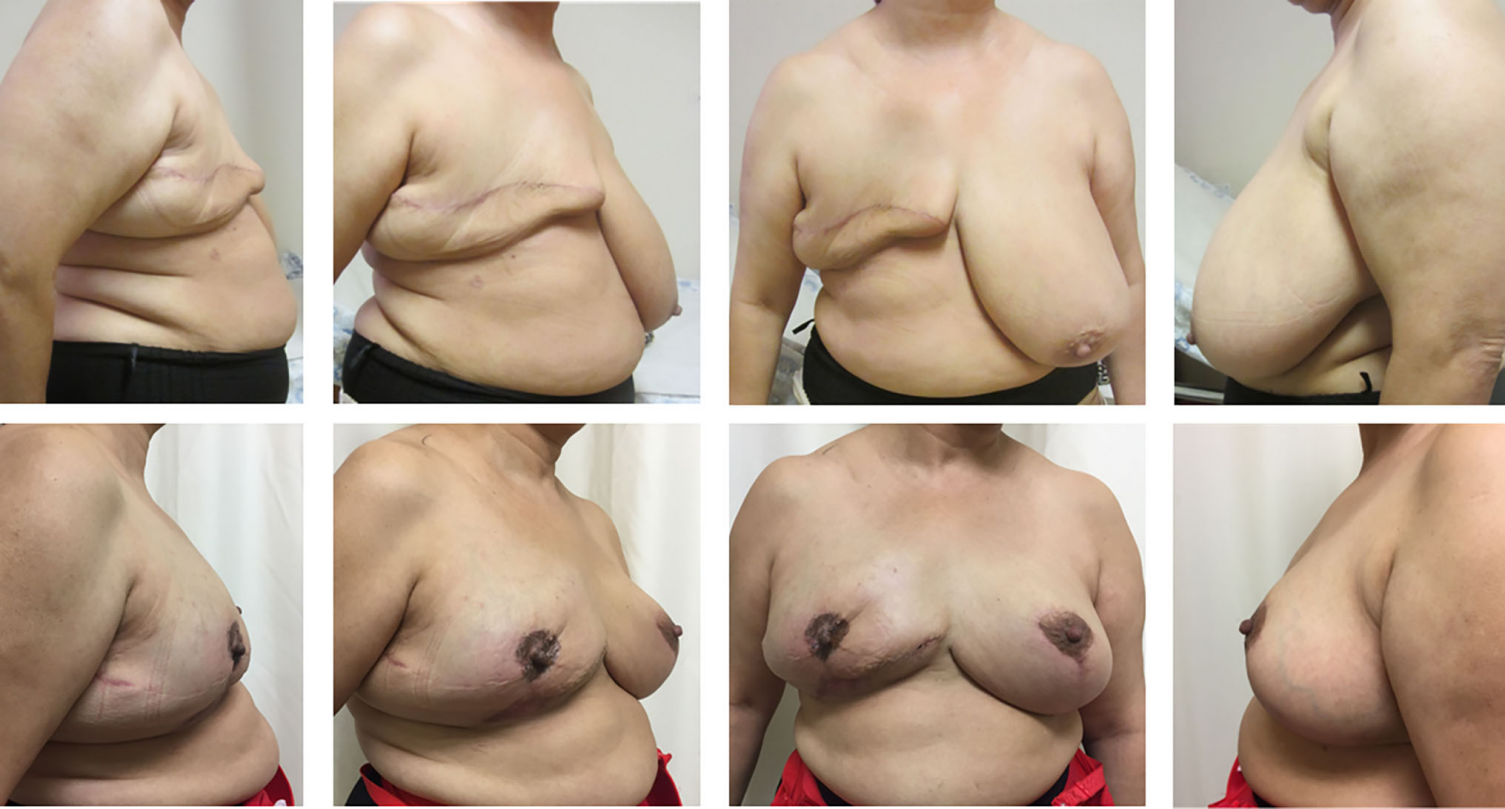
Above-after right mastectomy. Below-after 3 rounds of AFG to right breast (average of 240 cc per round), and conclusion with implant in neo-fat plane (Mentor, round Moderate-plus 550 cc) and left breast reduction

Between January 2015 and December 2022, a total of 29 patients (33 breasts) underwent delayed breast reconstruction with AFG and silicone implant as the final stage. The mean patient age was 43.5 ± 10.7 years (range, 30 to 55). The mean body mass index (BMI) was 29.6 ± 1.2 kg/m^2^ (range, 22.5 to 32.0 kg/m^2^) Three patients were BRCA1 carriers (10.3%), and five patients underwent post-mastectomy radiation therapy (15.2%). None received neoadjuvant radiation. Fourteen patients (48%) underwent chemotherapy, with nine receiving neoadjuvant and five receiving adjuvant treatment (Table 1). BRAVA was used in 15 patients (17 breasts, 51.5%) before each AFG. Patients had AFG between 1 and 5 times (mean= 3.4). All patients who underwent radiation therapy used the BRAVA and had AFG between 4 and 5 times (mean=4.4). Among other patients using the BRAVA, AFG occurred between 1 and 2 times (mean=1.5); while, those who did not use the BRAVA had AFG 2 and 5 times (mean=2.7). The mean autologous fat grafted at each session was 188 ± 21.5 cc (range, 150–240) for those who used the BRAVA and 140 ± 20.5 cc (range, 100–160) for the patients without BRAVA. The implants used had a mean volume of 320 ± 30 cc (range, 225–550). The mean total fat injected per patient was 470 cc (range, 400–780). The mean estimated fat take was 310 cc. In all cases, the final results were satisfying (Figures 1, 2). Patient’s satisfaction was 4.5 (range, 3.5–5). There were no major complications. Minor complications were observed with one patient and included an infection that resolved with oral antibiotics. (Table 2). Findings of fat necrosis and oil cysts occurred in 5 breasts (15.1%). All of them were determined by radiologists as small and benign, and did not require further investigation.

**Table 1:** Patient and oncologic characteristics

Characteristic	Value (%)
Total no. of patients	(100.0) 29
Total no. of breasts	33 (100.0)
Mean age ± SD (Range), years (*n* = 25)	5 ± 10.7 (30–55).43
Mean BMI ± SD (Range), kg/m2 (*n* = 25)	22.5–32.0)) 1.2 29.6 ±
Comorbidities	
Diabetes	2 (6.8)
Hypertension	1 (3.4)
Active smokers	4 (13.7)
BRCA carrier	3 (10.3)
Radiation therapy (breasts no.)	5 (15.2)
Neoadjuvant radiation therapy	0 (0.0)
Post-operative radiation therapy	5 (15.2)
Chemotherapy (patients no.)	14 (48.3)
Neoadjuvant chemotherapy	9 (31.0)
Post-operative chemotherapy	5 (17.0)

**Table 2:** Surgical Characteristics and Outcomes of Breasts

Characteristic	Value (%)
BRAVA usage	17 (51.5)
Unilateral	13 (39.4)
Bilateral	4 (12.1)
No. of AFG Sessions of Breasts without BRAVA	
1	0 (0)
2	5 (31.3)
3	7 (43.7)
4	2 (12.5)
5	2 (12.5)
Mean AFG Volume ± SD (Range) Breasts without BRAVA	140 ± 20.5 (100–160)
No. of AFG Sessions of Breasts with BRAVA	
1	6 (35.3)
2	6 (35.3)
3	0 (0.0)
4 [only radiated breasts]	3 (17.6)
5 [only radiated breasts]	2 (11.8)
Mean AFG Volume ± SD (Range) Breasts with BRAVA	188 ± 21.5 (150–240)
Mean Implant Volume ± SD (Range)	320 ± 25 (225–550)
Complications	
Hematoma	0 (0.0)
Infection	1 (3.6)
Seroma	0 (0.0)
Dehiscence	0 (0.0)
Necrosis	0 (0.0)
AFG-Autologous fat grafting	

## Discussion

The objective of the present study was to assess the effectiveness and patient satisfaction of advanced approach that involves implant insertion in a new fat plane, as a supplementary procedure to initial AFG in delayed breast reconstruction.

By focusing on enhancement of breast appearance, symmetry, and an overall aesthetic outcome in patients who have undergone mastectomy and delayed breast reconstruction, our study offers a unique perspective on the potential advantages of this approach. In conventional breast reconstruction, the sub-pectoral and pre-pectoral planes are commonly employed. The sub-pectoral plane offers improved implant coverage and reduced risk of visible rippling [25, 26]. However, it can lead to implant animation deformity and increased post-operative discomfort. On the other hand, the pre-pectoral plane provides a simpler procedure with reduced pain or discomfort and a better aesthetic result that eliminates animation deformity [27]. In delayed reconstruction, it is sometimes impossible to create a pre-pectoral pocket because of the quality of the tissue, the thinness of the skin or lack of sub-dermal thickness.

The use of AFG helps to improve all these problems and eliminates the need for ADM use, but usually a few grafting rounds are required to create the desired shape and size of the breast. BRAVA has proven to facilitate fat take by creating a better plane and promoting neovascularization. In our study, its usage was mandatory for radiated breasts. Incorporation of silicone implants as an adjuvant to AFG presents several potential advantages compared to using AFG alone. Firstly, the combination of implants and fat reduces the need for multiple fat grafting rounds, thereby decreasing treatment time and potential donor site morbidity. Furthermore, the presence of silicone implants provides a more predictable breast appearance as the implants contribute to the final shape and projection. Compared to relying solely on fat grafting, the adjunct use of implants yields more permanent outcomes, thereby achieving improved durability and stability. This combined approach has demonstrated its efficacy in improving breast appearance, symmetry, and aesthetic outcomes.

When considering the sequencing of procedures in delayed breast reconstruction, incorporating silicone implants after AFG offers several advantages over the reverse order. One key factor to consider is the impact of radiation therapy, which is commonly employed in the treatment of breast cancer. Radiation can have detrimental effects on the tissue, leading to decreased elasticity and compliance [28]. Performing AFG before the insertion of a silicone implant allows for the transfer of healthy and viable fat tissue to a vascular-competent breast area and, consequently, the rebuilding of damaged tissue. The addition of silicone implants allows for completion of the reconstruction process without necessitating additional sessions of fat grafting.

Throughout the course of our study and the follow-up period, no major complications were encountered, and no revision surgeries were required. A single patient developed a local infection that was treated conservatively. Findings of fat necrosis and oil cysts occurred in 5 breasts and were interpreted as small and benign, not requiring further investigation. Previous studies have shown a similar percentage of these findings after AFG, and there is common agreement that they can be detected as a consequence of AFG and do not cause any harm [28, 29].

Our patients expressed a high level of satisfaction with the outcomes at various follow-up periods. The results of our study emphasize the safety, efficacy, and versatility of this innovative technique in a diverse patient population. Patient’s satisfaction was very high.

## Conclusions

In conclusion, this study has explored an approach in delayed breast reconstruction that combines AFG followed by implant insertion into a new fat plane created by the grafting. The findings have demonstrated the effectiveness of this procedure together with patient satisfaction, thus highlighting the potential advantages that this approach offers over conventional techniques.

## Supplementary Information

Below is the link to the electronic supplementary material.

Supplementary file1 Technique of fat harvesting and grafting (MP4 81416 KB)

Supplementary file2 Technique of neo-fat plane creation before implant insertion (MP4 7183 KB)
